# Stabilisation dynamique d'un winging scapula (à propos d'un cas avec revue de la littérature)

**DOI:** 10.11604/pamj.2014.19.331.3429

**Published:** 2014-11-27

**Authors:** Jalal Boukhris, Mostapha Boussouga, Abdelouahab Jaafar, Nabil Bouslmame

**Affiliations:** 1 Service de Traumatologie Orthopedie II– Hmimv, Rabat, Maroc

**Keywords:** Scapula, stabilisation dynamique, muscle grand pectoral, Scapula, dynamic stabilization, pectoralis major

## Abstract

Décrit pour la première fois par Velpeau en 1937, le winging scapula reste une affection rare, encore peu connue aussi bien du grand public que des professionnels de santé. Il s'agit en fait de la paralysie isolée du nerf thoracique long, responsable de l'innervation unique du muscle serratus antérieur, laquelle paralysie génère un décollement du bord spinal et de la pointe de l'omoplate, particulièrement visible lors des mouvements d'abduction et d'antépulsion du bras. Evoluant habituellement vers la récupération spontanée, le diagnostic de cette affection est essentiellement clinique, l'exploration électromyographique, peut appuyer le diagnostic et surtout servir d’élément de surveillance. Le traitement est avant tout conservateur; la chirurgie n’étant envisagée que dans les formes chroniques qui ne répondent pas à la rééducation, le cas d'ailleurs de notre patient. Le choix du type d'intervention devra obéir à des critères précis. La stabilisation dynamique de la scapula est une intervention séduisante et donne entre des mains entraînées des résultats très satisfaisants, beaucoup de critiques sont faites sur la récupération de la force musculaire, ce qui en limite l'indication quand les exigences professionnelles des patients sont importantes. Néanmoins, certaines séries en font la méthode de choix avec des résultats excellents.

## Introduction

Le winging scapula est une pathologie rare, il se traduit par un décollement du bord spinal et de la pointe de l'omoplate à partir de la cage thoracique lors de l'antépulsion et de l'abduction du bras. Il est du à la paralysie isolée du nerf thoracique long dans diverses circonstances étiologiques. Pathologie handicapante sur le plan fonctionnel et esthétique, les avis quand à son traitement sont toujours partagés. Les traitements fonctionnels doivent toujours être proposés en première intention, la chirurgie n’étant envisagée qu'en cas d’échec de ces traitements, diverses méthodes sont rapportées, les indications de chacune ne font l'objet d'aucun consensus en raison de la rareté de la pathologie et de l'absence de séries chirurgicales dépassant exceptionnellement les cinq cas. Nous rapportons dans ce travail un cas de winging scapula diagnostiqué tôt, suffisamment traité par la méthode fonctionnelle sans résultats et stabilisé par un transfert du tendon du grand pectoral.

## Patient et observation

### Observation

**Figure 1:**
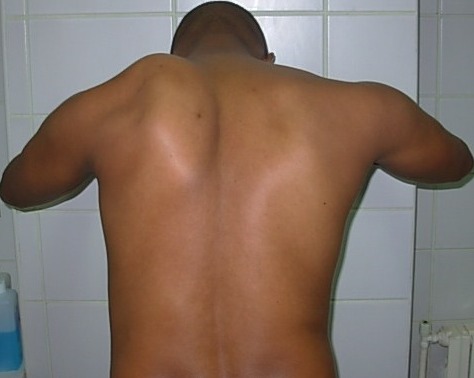
Exercice de pompes contre le mur matérialisant mieux le décollement du bord spinal de l'omoplate du côté attaint

Nous rapportons le cas d'un patient âgé 24ans, soldat très actif, musclé et sportif, sans antécédents pathologiques notables, s'est présenté à la consultation pour des douleurs scapulaires gauches d'allure inflammatoire, évoluant depuis 6 mois. Il avait également constaté depuis 3 mois, la perte de l'abduction et de l'antépulsion de l’épaule gauche au delà de 90°, son entourage ayant noté par ailleurs l'apparition d'une déformation manifeste sur la face postérieure de son épaule gauche, lorsqu'il effectuait des mouvements d'abduction et d'antépulsion. A l'inspection du dos on a constaté le décollement de l'omoplate provoqué par le patient, ce décollement s'exagère lorsqu'on demande au patient d'effectuer des pompes contre le mur (Figure 1). L'examen clinique a objectivé une limitation de l'antépulsion et l'abduction à 90°. Devant cet aspect clinique conforté par notre expérience précédente, nous avons soulevé voire posé le diagnostic de winging scapula, par paralysie du nerf thoracique long, que l'on a allégué chez le patient aux exercices physiques intensifs.

Afin de confirmer ce diagnostic, un bilan complémentaire a été demandé comportant un électromyogramme ayant conclu à une conduction nerveuse dans les limites de la normale, la détection musculaire objectivant un tracé comportant une activité neurogène critique dans les territoires C5- C6 ou tronc primaire. Ce résultat peu contributif, abondait néanmoins dans le sens du diagnostic de winging scapula. Le bilan radiologique standard a consisté en une radiographie de l'omoplate gauche de face et de profil, une radiographie thoracique ainsi que des radiographies du rachis cervical de face, de profil et de ¾, lequel bilan s'est révélé normal. L'avis des confrères neurologues et internistes, ainsi que le bilan biologique, permettait d’éliminer une pathologie générale dans le cadre de laquelle s'inscrirait la paralysie du nerf thoracique long. Devant les résultats de l'examen clinique, du bilan paraclinique, conforté par l'avis des confrères spécialistes, on a retenu le diagnostic de winging scapula par paralysie du nerf thoracique long en rapport avec les microtraumatismes répétés lors des exercices physiques et de musculation intensifs. Un traitement fonctionnel premier a été décidé, et le patient a été confié au kinésithérapeute. Il a ainsi bénéficié de 50 séances de rééducation étalées sur 06 mois. Au 6ème mois, l'examen clinique est resté inchangé, la douleur était aussi intense, bien qu'elle céda légèrement aux antalgiques et aux anti-inflammatoires non stéroïdiens sans jamais disparaitre complètement. Devant la douleur gênante et insomniante, l'handicap fonctionnel de plus en plus pesant et la gêne esthétique de plus en plus dérangeante, l'indication du traitement chirurgical a été retenue. Vu que le patient était jeune, qu'il avait une musculature particulièrement bien développée, et qu'il avait consulté tôt, permettant d’éliminer ainsi le risque de dégénérescence graisseuse de ses muscles; un traitement conservateur par stabilisation dynamique de la scapula lui a été proposé et nous avons opté pour la technique de transfert du tendon du grand pectoral rallongé par une bandelette du fascia-lata.

### Technique chirurgicale

L'intervention se déroule sous anesthésie générale, patient en décubitus dorsal, avec un coussin sous le moignon de l’épaule soulevant celle-ci, le membre supérieur gauche drapé en totalité et en abduction totale posé sur une tablette latérale. Le membre inférieur homolatéral est de même drapé en totalité pour pouvoir réaliser le prélèvement par la suite. L'abord fut via une incision oblique directe, en bas et en dehors à travers l'aisselle, du milieu du grand pectoral à la pointe de l'omoplate préalablement repérée à travers la peau.

**Figure 2:**
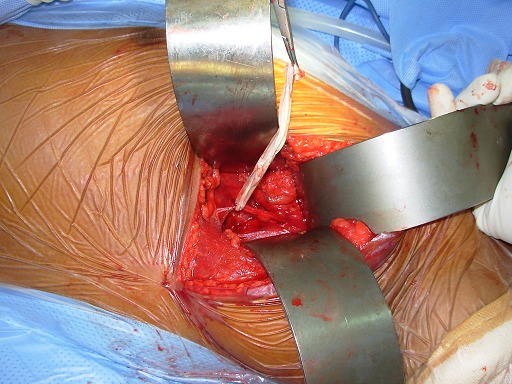
Isolement et neurolyse du nerf thoracique long le long de la cage thoracique

**Figure 3:**
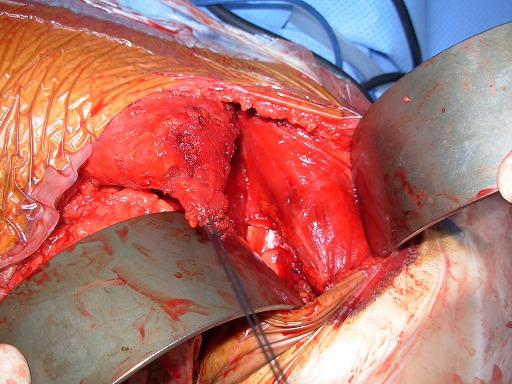
Individualisation et prélèvement de la moitié du tendon du grand pectoral

**Figure 4:**
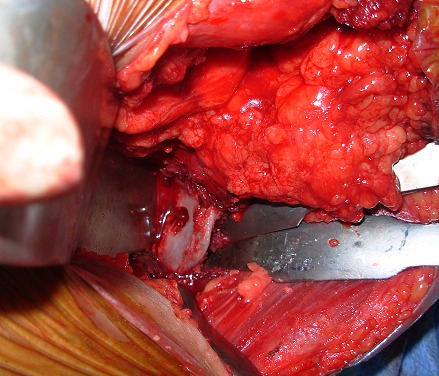
Squelettisation de la pointe de la scapula et confection d'un trou d’à peu prés 1.5cm× 1.5cm de diamètre au niveau de sa pointe

**1^er^ temps opératoire :** on repère le nerf thoracique long afin d'en faire la neurolyse la plus complète possible que permet la voie axillaire (Figure 2), cette neurolyse étant réalisée d'abord à visée antalgique, et non pas comme traitement du winging scapula. Le tendon du muscle grand pectoral étant individualisé, il est disposé sous forme de «U». La moitié du tendon est prélevée (Figure 3), la pointe de l'omoplate est ensuite squelettisée, permettant la confection d'un trou d'environ 1.5cm ×1.5cm de diamètre à ce niveau (Figure 4).

**2^ème^ temps opératoire:** on découvre la cuisse par une incision longue de 20 cm, puis l'on prélève une bandelette de la partie postérieure du fascia lata mesurant 20 cm× 3 cm. Cette bandelette du fascia lata est ensuite tubulisée en utilisant un fil non résorbable.

**Figure 5:**
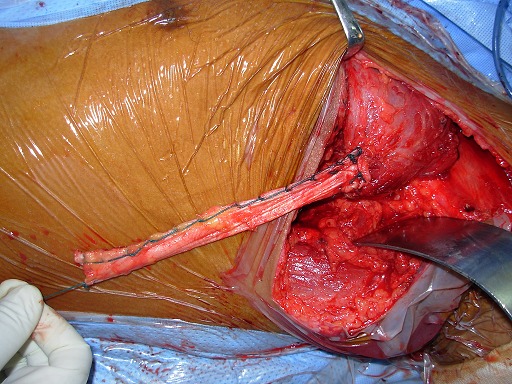
Suture de la bandelette du fascia lata à l'hémi-tendon du grand pectoral

**Figure 6:**
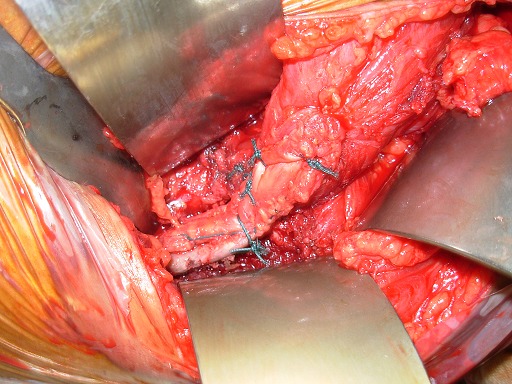
Suture de la bandelette à elle-même puis à l'hémi-tendon du grand pectoral après son passage dans le trou confectionné au niveau de la pointe de l'omoplate

**3^ème^ temps opératoire:** la bandelette du fascia lata est suturée à l'aide d'un fil non résorbable à l'hémi-tendon du grand pectoral (Figure 5). Elle est ensuite passée dans le trou confectionné au niveau de la pointe de l'omoplate. Le passage se fait d'avant en arrière, et de dedans en dehors pour ne pas provoquer un winging scapula stable iatrogène par la suite. La bandelette est ensuite retournée sur elle-même, suturée à elle- même et resuturée à l'hémi-tendon du grand pectoral (Figure 6). La fermeture se fait plan par plan sur un redon aspiratif. En post'opératoire, le patient est immobilisé dans un Dujarier coude au corps utilisant plusieurs bandes d’élastoplast.

**Le postopératoire:** L'immobilisation a été maintenue coude au corps pendant 45 jours. Le patient avait ressenti dés le 15^ème^ jour postopératoire une diminution progressive des douleurs lesquelles ont totalement disparu en un mois et demi, le sevrage de l'immobilisation a été alors entrepris et la rééducation a été entamée. Cette rééducation visait dans un premier temps la récupération des amplitudes articulaires en passif, ce qui a été obtenu en un mois et demi, ensuite la récupération de la mobilité active qui était totale a la fin du 4^ème^ mois postopératoire.

**Figure 7:**
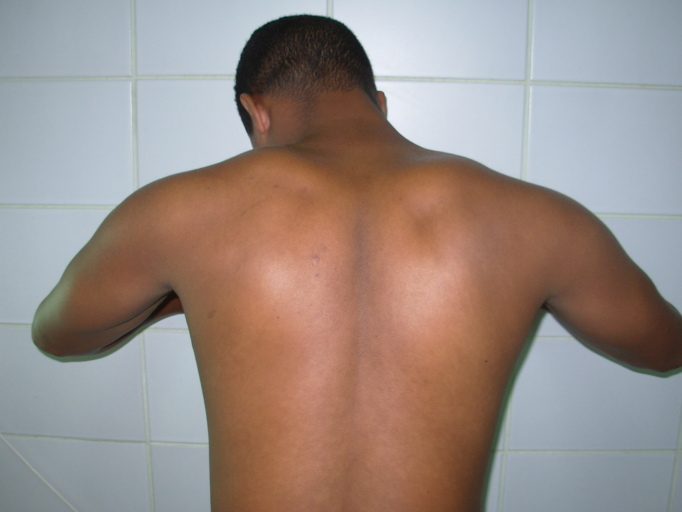
Manœuvre de traction contre le mur sans décollement de l'omoplate

**Résultats:** à la fin du 4^ème^ mois postopératoire, le résultat obtenu était excellent avec notamment une disparition totale des douleurs rapportées par le patient et l'obtention d'une antépulsion et d'une abduction complètes. La seule plainte relative du patient concernait la cicatrice opératoire jugée inesthétique bien qu'elle ne le gênait pas du tout puisqu'elle est enfouie dans le creux axillaire. Le patient était très satisfait du résultat (Figure 7).

## Discussion

Pour beaucoup d'auteurs [1–4], la stabilisation dynamique de la scapula correspond à la technique de choix chez tous les malades, notamment chez le sujet jeune et actif [4], après échec du traitement conservateur. Son avantage essentiel est qu'elle corrige le décollement scapulaire tout en maintenant une bonne mobilité articulaire scapulo-thoracique [5, 6] avec un résultat fonctionnel satisfaisant et une immobilisation post-opératoire plus courte. Le principal inconvénient semble être la perte de la force musculaire. Parmi les différentes transplantations musculaires possibles, celle du pectoralis major, semble la plus simple du point de vue technique, et la plus garante d'un bon résultat; c'est également la transplantation musculaire la plus répandue.

Fery [1] a rapporté 9 stabilisations par le pectoralis major dont 7 excellents résultats (disparition de toute symptomatologie) et 2 bons résultats (persistance d'un petit winging scapula à l'effort contre résistance). Iceton [7] a publié une série de 15 cas, de transfert du pectoralis major où les résultats ont été considérés comme bons chez 9 patients ayant une paralysie isolée du serratus antérieur; il recommande cette technique uniquement pour les atteintes isolées et la déconseille ailleurs où il préfère des arthrodèses scapulo-thoraciques. Connor et al. ont rapporté 11 patients avec des résultats satisfaisants chez 91% et une amélioration fonctionnelle significative ainsi qu'une réduction de la douleur [8]. Connor [9], Perlumutter [3], Post [10] et plusieurs autres, s'accordent pour dire que cette technique peut restituer une force musculaire convenable, mais chez les travailleurs lourds et chez les sportifs, le transplant est trop sollicité et la stabilisation dynamique ne semble pas être une bonne indication. Récemment, Noerdlinger et al [11] ont démontré lors d'une mise à jour d'une série antérieure qu'un soulagement total de la douleur et de la gêne fonctionnelle n’était pas toujours obtenu, malgré cela, la plupart des patients ont retrouvé leur niveau d'activité préopératoire avec des adaptations mineures. Malheureusement, de nombreux patients se sont plaints de la morbidité importante du site de prélèvement de l'autogreffe du fascia-lata.

L'inconvénient majeur est le prélèvement de la greffe de fascia-lata qui entraîne une large cicatrice sur la cuisse; le retentissement fonctionnel de cette prise de greffe sur la fonction du membre inférieur n'est pas évalué dans les différentes études [5]. Galano et al. [4] rapportent une récupération complète de la mobilité active chez 9 malades sur 11. Une amélioration significative de l'antépulsion moyenne (158,2°- 164,5°), des scores fonctionnels ASES (American Shoulder and Elbow Surgeons Shoulder) (de 53,3 à 63,8), et des échelles visuelles analogiques (de 5.0 à 2.9), étaient notées chez l'ensemble des patients. Le suivi a objectivé un lâchage du transfert dans un cas, 6 mois après l'intervention suite à un traumatisme. Les complications rapportées sont à type d'infections du site opératoire dans deux cas, ainsi qu'une asymétrie inesthétique des seins chez une patiente.

## Conclusion

A la lumière de nos résultats et ceux de la littérature, il parait clair que la stabilisation dynamique de la scapula est une intervention séduisante et donne entre des mains entraînées des résultats très satisfaisants, beaucoup de critiques sont faites sur la récupération de la force musculaire, ce qui en limite l'indication quand les exigences professionnelles des patients sont importantes. Notre cas n'est là que pour illustrer la pathologie et ouvrir le débat sur les indications opératoires; il ne nous permet de tirer aucune conclusion formelle. Néanmoins, il illustre l'efficacité de la méthode quand elle réussit, mais aussi ses aléas lorsqu'elle échoue. Nous espérons pouvoir présenter dans l'avenir, une série plus consistante comparant des résultats personnels.
